# Survival of Patients with Cholangiocarcinoma Receiving Surgical Treatment in an *O. viverrini* Endemic Area in Thailand: A Retrospective Cohort Study

**DOI:** 10.31557/APJCP.2020.21.4.903

**Published:** 2020-04

**Authors:** Satsawat Chansitthichok, Parinya Chamnan, Poowanai Sarkhampee, Nithi Lertsawatvicha, Pim Voravisutthikul, Paiwan Wattanarath

**Affiliations:** 1 *General Surgery Division, Department of Surgery, *; 2 *Cardiometabolic Research Group, Department of Social Medicine, Sanpasitthiprasong Hospital, Ubon Ratchathani, Thailand. *

**Keywords:** Survival, surgical outcome, cholangiocarcinoma, risk factors

## Abstract

**Objective:**

To investigate risk factors associated with mortality in cholangiocarcinoma patients receiving surgical treatment in Thailand’s endemic area and their survival rate.

**Materials and Methods:**

Medical records of patients with histologically confirmed cholangiocarcinoma, who underwent surgical treatment at Sanpasitthiprasong Regional Hospital from October 1, 2013 to October, 31 2015, were retrospectively included. Patients’ vital status (death/alive) and date of death were obtained from the Interior Ministry’s death certificate. Cox proportional hazard regression was used to examine factors associated with mortality.

**Results:**

Out of 295 patients with cholangiocarcinoma (CCA), 180(58%) were intrahepatic CCA, 86(28%) were perihilar CCA, and 29 (9%) were distal CCA. Three groups were homogenous in terms of age and gender. Most of our patients referred with abdominal pain (63%), especially those who were intrahepatic CCA (77%). However, almost 80% of the perihilar CCA and distal CCA patients came with jaundice. Tumor markers (CEA and CA19-9) were not different between groups p=0.74 and p=0.43 respectively. Median survival of patients with intrahepatic CCA, perihilar CCA, and distal CCA patients was 14.6, 14.2, and 14.0 months, respectively. Factors independently associated with mortality in intrahepatic CCA patients were number and size of tumors and presence of perineural invasion (Hazard ratio (HR) 1.09[1.03 - 1.15], 1.07[1.02 - 1.13], and 2.09 [1.28 - 3.39], respectively). In perihilar CCA patients, having positive lymph nodes and resection status were independently associated with mortality. Compared to R0 resection, R1, R2, and no resection of perihilar CCA were associated with a 2-, 8- and 4-fold increase in the risk of mortality (HR 2.17 (0.99 – 4.78), 7.97 (3.22 – 19.71), and 4.21 (0.51 – 34.82), respectively).

**Conclusion:**

CCA patients in this endemic area had fairly poor survival. Factors associated with mortality in intrahepatic CCA were number and size of tumors and perineural invasion. However, risk factors for perihilar CCA included positive lymph nodes and resection status.

## Introduction

Cholangiocarcinoma (CCA) is a malignancy originating from the biliary epithelium and peri-biliary gland. It can occur in every part of the biliary system ranging from the proximal small intrahepatic bile duct to the distal large common bile duct before entering the duodenum (Shaib and El-Serag, 2004; Blechacz and Gores, 2008). In Thailand, patients with CCA have a very poor prognosis with an overall median survival of 4 months after being admitted at a tertiary hospital (Luvira et al., 2016). The highest incidence of CCA in the world is reported in the north-eastern provinces of Thailand with 135.4 per 100,000 persons/year in men and 43.0 per 100,000 persons/year in women; whereas, its incidence is less than 1 per 100,000 persons/year in western countries (Khuntikeo et al., 2015; Squadroni et al., 2017).

According to previous studies, CCA cure is rare, with a 5-year survival ranging from 5% in intrahepatic CCA(iCCA) to 17% in extrahepatic CCA (Squadroni et al., 2017). Factors associated with survival rate of CCA after surgery include tumor size, number of tumors, lymph node metastasis, perineural invasion (PNI), lympho-vascular invasion (LVI) and marginal status (Yamamoto and Ariizumi, 2011; Song et al., 2013; Hartog et al., 2016; Bagante et al., 2017; Jeong et al., 2017; Squadroni et al., 2017; Wellner et al., 2017). However, most of these studies were done in developed countries where the natural history of CCA and its treatment may differ from those in developing countries where there are other causative factors, such as infection with the liver fluke, Opisthorchis viverrini. Little data exists on survival of patients with CCA and its predictors in developing countries, and most studies had small sample sizes and were carried out decades ago (Uttaravichien and Buddhisawasdi, 1990; Uttaravichien et al., 1999). 

Therefore, the present study aimed to examine survival rates and factors associated with survival in a contemporary cohort of CCA patients surgically treated at a regional tertiary hospital in northeast Thailand. The information presented may be useful for physicians in selecting cases for surgery or adjuvant chemo-radiation therapy.

## Materials and Methods

*Design overview*

This retrospective cohort study based on data from 334 bile duct tumor patients treated at the Department of Surgery, Sanpasitthiprasong Regional Hospital, Ubon Ratchathani, between October 1, 2013 and October 31, 2015. Medical records of patients with histologically confirmed bile duct tumors undergoing surgical exploration were reviewed. Data on personal and medical history, symptoms and signs, laboratory and radiological findings, as well as operative and pathological reports were obtained. These data were then linked to mortality data from the Ministry of Interior’s death certificates. We excluded 23 patients with incomplete data regarding the above-mentioned variables and foreigners whose vital status could not be confirmed, leaving a final study population of 311 patients.

*Medical evaluation and surgical treatment*

Patients were divided into four groups according to the site of the primary tumor and its characteristics as follows: 1) Intrahepatic CCA (iCCA), 2) Perihilar CCA (pCCA), 3) Distal CCA (dCCA), and 4) other bile duct neoplasms. Both the iCCA and pCCA subgroups were defined by their tumor characteristics based on radiological, operative, and pathological findings. iCCA was divided into mass-forming (mf), periductal-infiltration (pi), intraductal growth (id), and mixed type. pCCA was divided into nodular (nod), sclerosing (scl; periductal infiltration pattern at hilar region), and papillary (pap) types (Khan et al., 2005; Yamamoto and Ariizumi, 2011; Razumilava and Gores, 2014).

All patients underwent preoperative radiological examination, dynamic CT, and/or MRI imaging of the upper abdomen, and the results were interpreted by certified radiologists. All pathological specimens were examined by certified pathologists.

Our current surgical strategy was to achieve R0 resection, to perform hilar resection in every pCCA and iCCA invading the 1st order duct, and to perform node dissection in iCCA with clinical lymphadenopathy and in every pCCA case. We classified surgical interventions into curative intent, R2 resection, palliative bypass, and open and biopsy. Curative intent composed of R0 and R1 resection depended on the pathological outcomes; that is, whether tumor cells were present on the resection margin or not. R2 resection was attempted in young and healthy patients with vascular invasion, bi-lobar lesion, beyond locally lymph node metastasis (Station 8, 12, and 13), and frozen hepatoduodenal ligament or local peritoneal metastasis. Left intrahepatic duct hepaticojejunostomy was performed on all of the bypass group with iCCA and pCCA. The decision to choose intervention relied on the patients’ status, tumor characteristics, and the surgeon’s opinion. In this study, we used the AJCC 7th edition to determine final disease staging (Shindoh and Vauthey, 2014). After surgery, patients received chemo-radiation therapy if it was not a R0 resection, there were positive pathological lymph nodes, and recurrent disease. In this study, we used mortality at >90 days as a long-term survival outcome (Mayo et al., 2011). 

*Data collection*

Personal and medical history, symptoms and signs, laboratory (liver function test and tumor markers [CEA and CA 19-9]) and radiological findings (CT scan or MRI), and operative and pathological reports were obtained from the medical records of CCA patients. 

*Outcome ascertainment*

Vital status (deceased or alive) and dates of death for patients were obtained from the Ministry of Interior’s death certificates. Survival time was defined as the period from the date of surgery to the date of death, or to 10th April 2017 for those who survived. 

*Data analysis*

Statistical analyses were performed using SPSS, version 10.1 (Statistical Package for the Social Sciences, Chicago, IL). Patient characteristics were described as number (%) and mean (±SD) for categorical and continuous variables, respectively. Comparisons of these characteristics across groups were performed using chi-square test and ANOVA for categorical and continuous variables, respectively. The present study focused solely on long-term survival, excluding those who died within 90 days after surgery (perioperative mortality). Survival analyses were carried out separately for each CCA subgroup. Survival probabilities were estimated using the Kaplan-Meier method and compared by the log-rank test. Median survival (interquartile range, IQR) in months was reported. Univariate and multivariate Cox proportional hazard regression was used to identify factors associated with mortality in these patients. Factors associated with mortality in univariate regression (p<0.1) were included in the multivariate regression. A p-value of 0.05 was considered statistically significant.

## Results

Between October 1, 2013 and October 31, 2015, there were 570 major hepato-pancreato-biliary cases. Among these, 216 cases did not have bile duct tumors (HCC, colorectal cancer liver metastasis, pancreatic tumor, intrahepatic duct stone, and chronic inflammation and infection). These patients were excluded from the analysis. We further excluded 20 cases of ampulla adenocarcinoma and 23 cases who were foreigners and whose medical records were missing, leaving a final study population of 311 patients.

The characteristics of the study participants are shown in Table 1. The mean age (±SD) of the patients was 60.7 (9.1), with approximately two-thirds being male. Abdominal pain and jaundice/cholangitis were the most common presentations (63% and 40% of the patients, respectively). A total of 183 (59%) patients received curative surgery. None of our CCA cases had cirrhosis. All four tumor subgroups were of comparable age. The majority of cases were admitted with iCCA (57.9%). Most of the iCCA patients presented with abdominal pain, while almost 80% of the pCCA and dCCA patients presented with jaundice. Twenty-four (50%) patients with mixed type iCCA presented with obstructive jaundice. iCCA had the highest prevalence of distant metastasis detected. The other group was composed of three cases of biliary intraepithelial neoplasia, eight cases of IPMN-BT, two cases of biliary cystadenoma, and three cases of biliary cystadenocarcinoma. 

Table 2 shows the pathological results of 295 patients in the three different CCA tumor subgroups. Most of the tumors were moderately differentiated, except for nod- and pap-pCCA. There was a multi-nodular appearance only within the mf and mixed types of iCCA (22.4% and 9.7%, respectively). The median size of the mass lesion (IQR) in iCCA was 6.5 (5-9) cm and the mean was 7.4 cm. The largest tumor was observed in the mf and mixed types, with a mean size of 8.2 cm and 7.2 cm, respectively. There were 84 patients whose tumors were larger than 5 cm, and 31 whose tumors were larger than 10 cm in size. Most of the R2 resections in iCCA were observed in the mixed type 13(42%), and most R2 resections in pCCA were observed in the scl type 13(28.3%). Positive lymph nodes were found frequently in the mixed type iCCA, nod and scl pCCA, and dCCA. 

More than half of our patients with iCCA and pCCA presented with advanced tumor stages (stage III and IV), while only one-third of patients with dCCA had advanced stages at diagnosis (See Supplementary Table 1). 

The overall mortality of patients with CCA after surgical resection was 4.53 per 100 person-months, with different mortality of 4.53, 4.59 and 4.34 per 100 person-months for iCCA, pCCA and dCCA, respectively. The overall median (IQR) survival was 14.3 (7.7-22.1). The median (IQR) survival after surgical resection for iCCA, pCCA, and dCCA was 14.6 (8.4-22.2) months, 14.2 (7.4-22.4) months, and 14.0 (6.0-19.1) months, respectively. 

**Figure 1:**
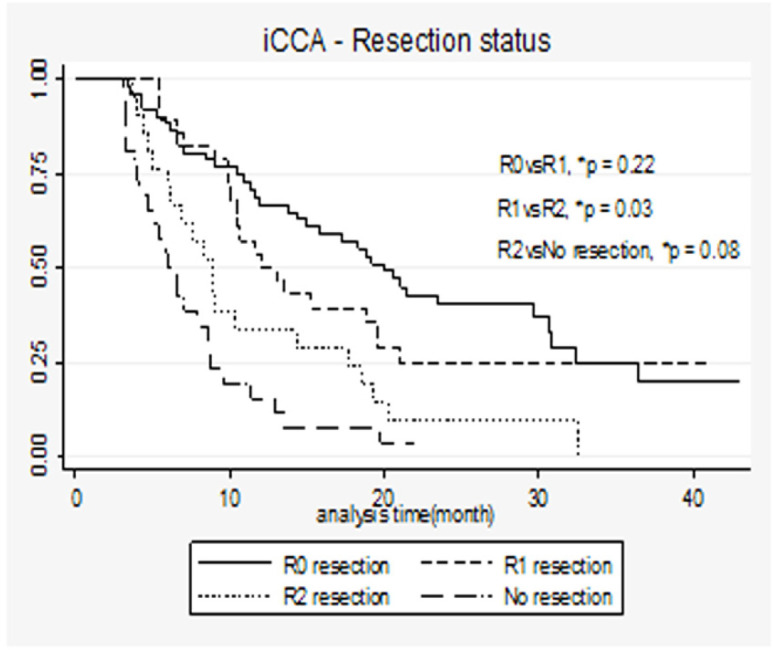
K-M Curves Demonstrating Long Term Survival of Patients with iCCA by Resection Status and Log-Rank Test (n=180). *P-value for comparison across groups using log-rank test

Figure 1 shows that survival status differed with respect to resection status. Survival varied across resection status (log rank p <0.001). The median survival of R0 in the iCCA group was 20 (10.5-29.2) months, while median survivals of R1, R2, and No Resection were 12.5 (9.8-20.9), 8.9 (6.0-17.6), and 6.3 (4.0-8.8) months, respectively. R0 had the best survival, but this was not statistically significantly different from R1. R2 resections had a slightly better survival outcome than no-resection group, but with no statistical significance.

**Figure 2:**
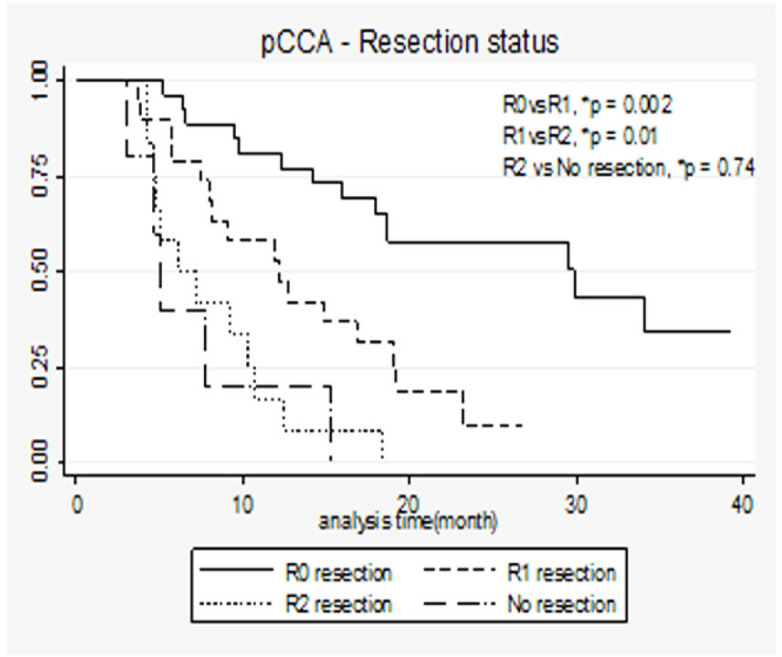
K-M Curves Demonstrating Long Term Survival of Patients with pCCA by Resection Status and Log-Rank test (n=86). *P-value for comparison across groups using log-ranked test

Similar findings were observed in patients with pCCA (Figure 2). That is, the survival rate varied with respect to resection status, patients with R0 clearly having better survival than those with R1 and R1 patients having better survival than those with R2 or no resection. The median survival of R0 in the pCCA group was 21.4 (14.2-29.8) months, while the corresponding figures for R1, R2, and no resection were 12.1 (7.4-19.1), 6.6 (4.7-10.5), and 5.0 (4.7-7.7), respectively. 

Table 3 shows factors associated with mortality after 90 days in patients with iCCA. Analyses by univariate Cox proportional hazard regression revealed factors associated with risk of death after 90 days in patients with iCCA were tumor characteristics, resection status, number and size of tumor (s), lympho-vascular invasion, perineural invasion, and positive lymph nodes (Table 3). However, other factors such as age, gender, levels of pre-operative bilirubin, and tumor markers were not associated with long-term mortality. Neither pathological grading, nor positive margin showed a significant association with the risk of mortality.

Based on multivariate Cox regression, factors independently associated with the risk of mortality in iCCA included resection status, number and size of the tumor (s), and PNI. Compared to patients with R0, those with R2 had more than a 2-fold increased risk of mortality (HR 2.09, 95% CI (1.18 - 3.73), p= 0.01), while R1 patients had a comparable risk. Risk of mortality increased 7 % for every 1 cm increase in tumor size. Every additional tumor was associated with a 9% increase in risk of mortality (HR 1.09, 95% CI (1.03 - 1.15), p= 0.002). Those with PNI had a 2-fold higher risk of mortality than those without (HR 2.09, 95% CI (1.28 – 3.39), p=0.003). These hazard ratios did not alter after controlling for other factors (Table 3). 

Regarding perihilar cholangiocarcinoma, we excluded papillary type from the univariate and multivariate cox analyses as no death occurred in this group. The only two factors associated with the risk of death based on multivariate cox regression were resection status and having positive lymph nodes (Supplementary Table 2). Compared to R0 resection, R1, R2, and no resection were associated with a 2-, 8-, and 4-fold increase in the risk of death (HR 2.17 (0.99 – 4.78), 7.97 (3.22 – 19.71), and 4.21 (0.51 – 34.82), respectively. Patients with positive lymph nodes were almost 3-times more likely to die than those without positive lymph nodes (HR 2.87 (1.45 – 5.70)). Tumor characteristics, number and size of tumor, pathological grading, presence of lympho-vascular, and perineural invasion were not associated with mortality risk in pCCA.

In terms of dCCA, median survival of the R0 group was 14 (8.8, 19.1) months. R0 patients had a slightly worse outcome compared to those with R1 resection without statistical significance, and patients with R2 had the worst survival outcome. All cases in the no-resection group had a palliative bypass. The only statistically significant outcome of dCCA based on univariate analysis was positive lymph node 6.86 (1.49 – 31.57)

**Table 1:** Demographic, Clinical Characteristics and Surgical Intervention in 311 Patients with Bile Duct Tumor by Tumor Subgroups

	Total	iCCA	pCCA	dCCA	Others	*P*-value
No. of patients (%)	311 (100)	180 (57.9)	86 (27.7)	29 (9.3)	16 (5.1)	
Mean age (SD)	60.7 (9.1)	60.3 (9.2)	61.3 (9.5)	62.9 (7.8)	57.5 (7.6)	0.23
Gender(%male)	218 (70.1)	120 (66.7)	70 (81.4)	19 (65.5)	9 (56.3)	0.05
Clinical presentation (%)						
Abdominal pain	194 (63.4)	138 (76.7)	34 (39.5)	12 (41.4)	10 (62.5)	<0.001
Jaundice/Cholangitis	125 (40.2)	32 (17.8)	69 (80.2)	22 (75.9)	2 (12.5)	<0.001
Weight loss	13 (4.2)	9 (5)	2 (2.3)	2 (6.9)	0 (0)	0.52
Accidental finding	7 (2.25)	6 (3.3)	0 (0)	0 (0)	1 (6.3)	0.19
Screening	20 (6.4)	10 (5.6)	5 (5.8)	1 (3.5)	4 (25)	0.02
Laboratory, mean (SD)						
Pre-op Bilirubin	7.5 (10.2)	3.3 (7)	14.9 (10.4)	15.4 (11.9)	0.5 (0.2)	<0.001
Pre-op Bilirubin if Jaundice	16.6 (9.7)	13.6 (9.4)	17.3 (9.2)	19.9 (9.9)	-	0.01
CEA(ng/dl)	63.3 (419)	61.2 (184)	101.1 (754)	3 (2)	2.1 (1.5)	0.74
CA19-9(U/ml)	1,398 (2715)	1,342 (2465)	1,573 (3183)	1,856 (3250)	424 (1542)	0.43
Surgical intervention (%)						<0.001
Curative intent(R0/R1)	183 (58.9)	92 (51.1)	55 (64)	21 (72.4)	15 (93.8)	
R2 resection	55 (17.7)	35 (19.4)	16 (18.6)	3 (10.3)	1 (6.2)	
Bypass	24 (7.7)	9 (5)	10 (11.6)	5 (17.2)	0 (0)	
Open and biopsy	49 (15.8)	44 (24.4)	5 (5.8)	0 (0)	0 (0)	

For comparisons across different tiers a chi-square test and ANOVA were used for categorical and normally-distributed continuous variables, respectively.

**Table 2:** Pathological Outcomes in 3 Subgroups of CCA (n=295 Patients).

	iCCA				pCCA			dCCA
	mf	pi	id	mixed	nod	scl	pap	dCCA
Grading (%)								
Well differentiation	16 (20.8)	5 (41.7)	2 (28.6)	11 (35.5)	11 (57.9)	18 (38.3)	4 (66.7)	9 (37.5)
Moderate differentiation	53 (68.8)	7 (58.3)	5 (71.4)	16 (51.6)	6 (31.6)	26 (55.3)	2 (33.3)	13 (51.2)
Poorly differentiation	8 (10.4)	0 (0)	0 (0)	4 (12.9)	2 (10.5)	3 (6.4)	0 (0)	2 (8.3)
No. of tumor (%)								
1	52 (68.4)	12 (100)	6 (85.7)	24 (77.4)	16 (84.2)	47 (100)	5 (88.3)	24 (100)
2	5 (6.6)	0 (0)	1 (14.3)	2 (6.5)	3 (15.8)	0 (0)	1 (16.7)	0 (0)
3	1 (1.32)	0 (0)	0 (0)	1 (3.2)	0 (0)	0 (0)	0 (0)	0 (0)
4	1 (1.32)	0 (0)	0 (0)	1 (3.2)	0 (0)	0 (0)	0 (0)	0 (0)
multiple	17 (22.4)	0 (0)	0 (0)	3 (9.7)	0 (0)	0 (0)	0 (0)	0 (0)
Mean size in cm (SD)	8.2 (4.3)	3.7 (1.3)	4 (1.1)	7.2 (2.5)	4.2 (1.5)	3.8 (1.9)	1.8 (1.7)	2.8 (1.5)
Positive margin (%)	30 (39)	2 (16.7)	2 (28.6)	26 (83.8)	9 (47.4)	31 (66.0)	1 (16.7)	5 (20.8)
Resection status								
R0 (%)	37 (48.1)	10 (83.3)	5 (71.4)	3 (9.7)	8 (42.1)	16 (34.8)	5 (83.3)	18 (75)
R1 (%)	18 (23.4)	2 (16.7)	2 (28.5)	15 (48.4)	8 (42.1)	17 (37.0)	1 (16.7)	3 (12.5)
R2 (%)	22 (28.6)	0 (0)	0 (0)	13 (41.9)	3 (15.8)	13 (28.3)	0 (0)	3 (12.5)
LVI (%)	39 (50.7)	1 (8.3)	1 (14.3)	24 (75)	13 (68.4)	30 (62.5)	1 (16.7)	10 (41.7)
PNI (%)	32 (41.6)	3 (25)	0 (0)	21 (65.6)	13 (68.4)	20 (41.7)	0 (0)	12 (50)
Node dissection (%)	40 (52.6)	4 (33.3)	2 (28.6)	26 (81.3)	19 (100)	42 (87.5)	6 (100)	24 (100)
Positive lymph node (%)	27 (35.5)	2 (16.7)	0 (0)	20 (62.5)	14 (73.7)	22 (45.8)	1 (33.3)	15 (62.5)

data are presented as number (%) and mean (SD) for categorical and continuous variables, respectively.

**Table 3:** Factors Associated with Long Term Mortality (>90 days) in Patients with iCCA, Using Univariate and Multivariate Cox Regression (n=180).

	Univariate analysis		Multivariate analysis	
	Hazard ratio [95%CI]	p-value	Hazard ratio [95%CI]	*P*-value
Sex (male as Ref.)	0.92 [0.60 - 1.39]	0.68	NA	
Age	1.00 [0.98 - 1.02]	0.99	NA	
Characteristic				
MF	Ref.			
PI	0.36 [0.15 - 0.74]	0.01	NA	
ID	0.18 [0.04 - 0.75]	0.02	NA	
Mix	1.21 [0.75 - 1.94]	0.79	NA	
Resection status				
R0 resection	Ref.		Ref.	
R1 resection	1.37 [0.79 - 2.36]	0.26	1.14 [0.64 - 2.23]	0.67
R2 Resection	2.61 [1.50 - 4.57]	0.001	2.09 [1.18 - 3.73]	0.01
No resection	4.70 [2.74 - 8.03]	<0.001	NA	
Laboratory				
Bilirubin	1.04 [0.99 - 1.08]	0.11	NA	
CEA	1.001 [0.999 - 1.002]	0.17	NA	
CA 19-9	1 [0.999 - 1.000]	0.34	NA	
Pathological outcome				
No. of tumor	1.12 [1.04 - 1.20]	0.004	1.09 [1.03 - 1.15]	0.002
Size(cm)	1.09 [1.04 - 1.14]	<0.001	1.07 [1.02 - 1.13]	0.01
Grading	1.27 [0.86 - 1.85]	0.22	NA	
Positive margin	1.45 [0.92 - 2.30]	0.11	NA	
Lympho-vascular invasion	2.1 [1.31 - 3.38]	0.002	NA	
Perineural invasion	2.1 [1.32 - 3.35]	0.002	2.09 [1.28 - 3.39]	0.003
Positive Lymph node	1.91 [1.19 - 3.06]	0.007	NA	

Hazard ratios were not altered after controlling for age, sex, bilirubin level, CEA, CA19-9.

## Discussion

In this large retrospective cohort of contemporary Thai patients with CCA, it was found that the survival rate was poor to moderate with varying survival rate with respect to resection status. In general, patients with iCCA showed a slightly higher survival than those with pCCA or dCCA. Factors independently associated with the risk of mortality in iCCA were resection status, number and size of tumor (s), and PNI. Factors associated with the risk of mortality in pCCA were resection status and positive lymph nodes, while the only factor associated with mortality in dCCA was the presence of positive lymph nodes. 

Surgical outcome in our study was poor compared to that in developed countries. Median survival (IQR) after surgical resection in our iCCA, pCCA and dCCA patients was 14.6 (8.4-22.2) months, 14.2 (7.4-22.4) months, and 14.0 (6.0-19.1) months, respectively. While in developed countries IQR in iCCA, pCCA, and dCCA patients were 15-40 months, 12-24 months, and 18 months, respectively (Olnes and Erlich, 2004; Yamamoto and Ariizumi, 2011; Lad and Kooby, 2014). Most of CCA patients in northeast Thailand come from low socioeconomic status groups and have low level awareness about the disease (Khuntikeo et al. unpublished data); hence our patients usually came with advanced stage or very large size tumors. Moreover, our universal health coverage package provided adjuvant treatment only in unresectable and distant metastasis cases. Some of our patients could not afford to get adjuvant or chemo-radiation therapy even if they had very poor prognosis factors. Univariate analysis of iCCA revealed that, some characteristics (pi and id) had a favourable survival outcome, which is line with results of a previous study (Yamamoto and Ariizumi, 2011).

Similar to previous studies, we could detect factors that can predict survival rate in patients with CCA (de Jong et al., 2011; Brown et al., 2014; Razumilava and Gores, 2014). In patients with iCCA, factors independently associated with survival rate included resection status, PNI, number of tumors, and and size of tumor (s). However, in our study we did not observe any impact of resection R1 on survival rate compared to R0, iCCA differentiation, lymph node metastasis, and LVI , which is in line with findings reported in studies from Europe by DeOliveira et al., (2007) and de Jong et al., (2011). In this study, R1 compared to R0 resection and differentiation in iCCA failed to show any statistically significant effect on survival rate (there must be some much stronger factor(s) affecting survival rate) (DeOliveira et al., 2007; de Jong et al., 2011). While lymph node metastasis and LVI gave a universally bad prognosis, these factors were only significant in the univariate analysis (DeOliveira et al., 2007; de Jong et al., 2011). Following multivariate analysis of iCCA, it was found that only resection status, PNI, number of tumor(s), and tumor size proved to be strong independent factors. According to Table 2, mf and mixed types (the majority of our iCCA) were likely to have worse prognostic factors compared to other characteristics in the iCCA group, which can be attributed to large size of the tumors. However, this result needs further study to be confirmed. 

In our univariate analysis for pCCA, we showed that resection status, marginal status, positive lymph nodes, and CA 19-9 level affected survival outcome, as in previous studies (Tyson and El-Serag, 2011; Razumilava and Gores, 2014; Zaydfudim et al., 2014; Hartog et al., 2016). Following multivariate analysis; however, it was revealed that only resection status and positive lymph nodes were strong independent factors. 

Previously, we assumed that R2 resection in advanced cases, such as distant metastases, and receiving adjuvant therapy may have been an advantage for some patients. However, in this study, it was found that it had very few benefits for iCCA cases and even increased risk in pCCA cases. This might be explained by the criteria used for case selection and should be explored.

Our study is among only a few studies that investigated the association between PNI and mortality (Fisher et al., 2012). Our results suggested a strong association between these two variables. The first possible explanation for this association is that PNI is the parameter showing how aggressive the tumor is and which tumor is likely to invade or metastasize to other tissues. Secondly, the larger size of the tumor can lead to a higher chance of bad prognosis. In this study, ANOVA analysis showed that large tumors had a higher chance of invading peripheral tissue (p = 0.010). Thirdly, we do not routinely perform lymph node dissection in iCCA (hepato-duodenal ligament skeletonization or node sampling was performed on 57% of iCCA). In some cases, patients might have sub-clinical lymph node metastases, but biopsies are taken from every case that underwent resection to determine perineural invasion. Additionally, PNI is a parameter determining how aggressive the tumor is. With PNI, the tumor is likely to invade or metastasize to other tissues.

This study provided a basis to improve the criteria with which we can select patients to receive a screening program and adjuvant therapy. During the period of study, we performed ultrasonography in people from our region. The inclusion criteria were, Thai 40 years of age or over with any of the following: ever been infected by or treated for liver flukes or known to have eaten uncooked freshwater fish with scales (Khuntikeo et al., 2015). The screening program which involved ultrasonography screening in more than 62,000 patients led to the diagnosis of only 20 CCA patients who were treated in our hospital. This was the only hospital capable of treating CCA at that time from our area, and the universal health coverage program would not allow patients to be treated from outside of the area. Therefore, the benefits of such a program in our opinion are uncertain and need to be investigated using a more rigorous study design. We suggest adding additional clinical presentation selection criteria to improve the benefit of the program (Khuntikeo et al., 2015). For example, instead of performing ultrasonography for all people fitting the criteria, we suggest a focus only on individuals with unexplainable abdominal pain, untreated dyspepsia, or unexplainable weight loss. 

Our study was one of largest single-site studies in Asia and it presented clinical characteristics and outcomes of a contemporary population of CCA patients, with long-term mortality data verified using national death certificates. Excluding perioperative deaths, our study exclusively described disease-related mortality in the region with the world’s highest CCA incidence. However, our study had a number of limitations. The completeness and accuracy of some clinical data might be limited due to its retrospective study design. However, most key clinical variables were complete, and these were adjusted in the regression models. The relatively small number of patients with pCCA and dCCA may have resulted in reduced power to detect the association between certain factors and survival rate. A prospective data collection from these groups of patients and a multi-site study approach may help enhance the possibility of identifying additional predictors, including novel biomarkers. Generally, there were too few cases with pCCA and dCCA. Moreover, we focused only on long-term survival rate (<90 days mortality). 

In conclusion, even though the success of our CCA treatment still cannot compare to that of developed countries, the situation of CCA patients in our region is not as desperate as it was a few decades ago. Our study showed that the outcome of treatment depends on the size and number of tumors and achieving an R0 resection (pCCA group). This result could be improved by increasing the awareness of our patients about this disease so that they can be treated while the disease is still at an early stage. 
